# Supplementary material for: Evidence for magmatic carbon bias in 14C dating of the Taupo and other major eruptions
Source: Nat Commun. 2018 Oct 5;9:4110. doi: 10.1038/s41467-018-06357-0 (PMC6173711; doi:10.1038/s41467-018-06357-0)
Supplement: Supplementary file 1 — Supplementary Information [file 41467_2018_6357_MOESM1_ESM.pdf]

Supplementary Information

Evidence for magmatic carbon bias in ^{14}C dating of the Taupo and other major eruptions

Holdaway *et al*.

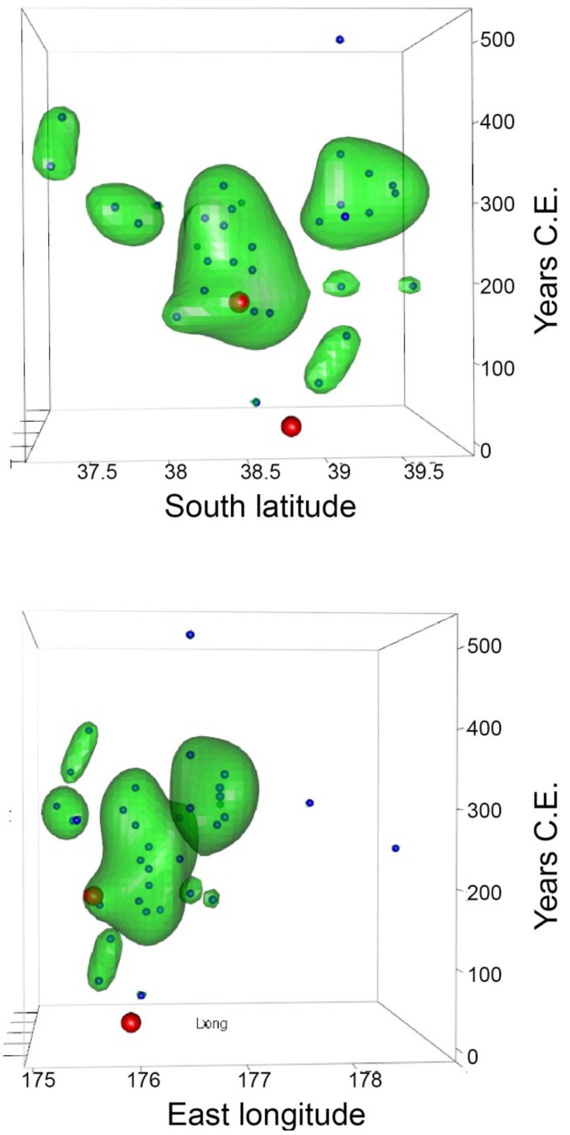

Supplementary Figure 1 Radiocarbon ages on materials from close to the vent of the First Millennium eruption of Taupo volcano are older than those on samples from further away. 3D Kernel distributions of median calibrated radiocarbon ages. calculated using packages ks, misc3d, and rgl in R Version 3.3.3. Green envelopes are 95.4% kernel density surfaces. Large symbols: lower, vent; upper (within envelope), wiggle match tree outer rings date.

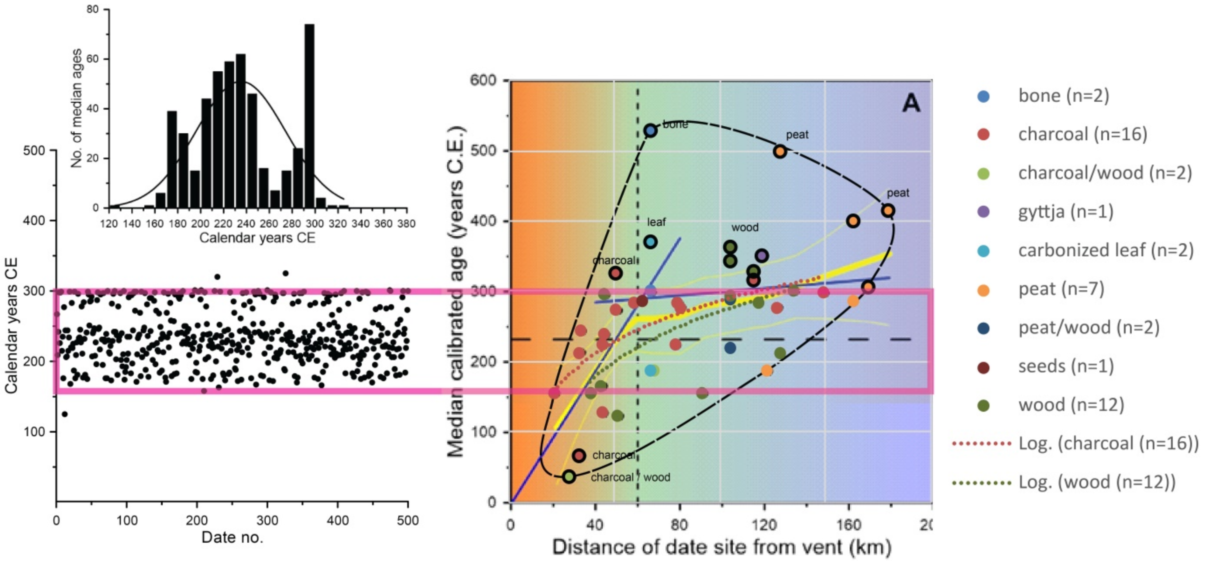

Supplementary Figure 2 Distribution (at left) of median calibrated dates for 500 randomly generated radiocarbon ages for 232 CE, modelled with 25-year measurement errors, using the SHCal13 curve. Note that only two of the 500 random dates exceed 300 CE and that the distribution does not include the corpus of young ages on samples from > 60 km from the vent. Colour codes for dated materials at right. Histogram of random dates exhibits the peaks of the calibration curve, but the scatter does not include the "young" ages discussed in this paper.

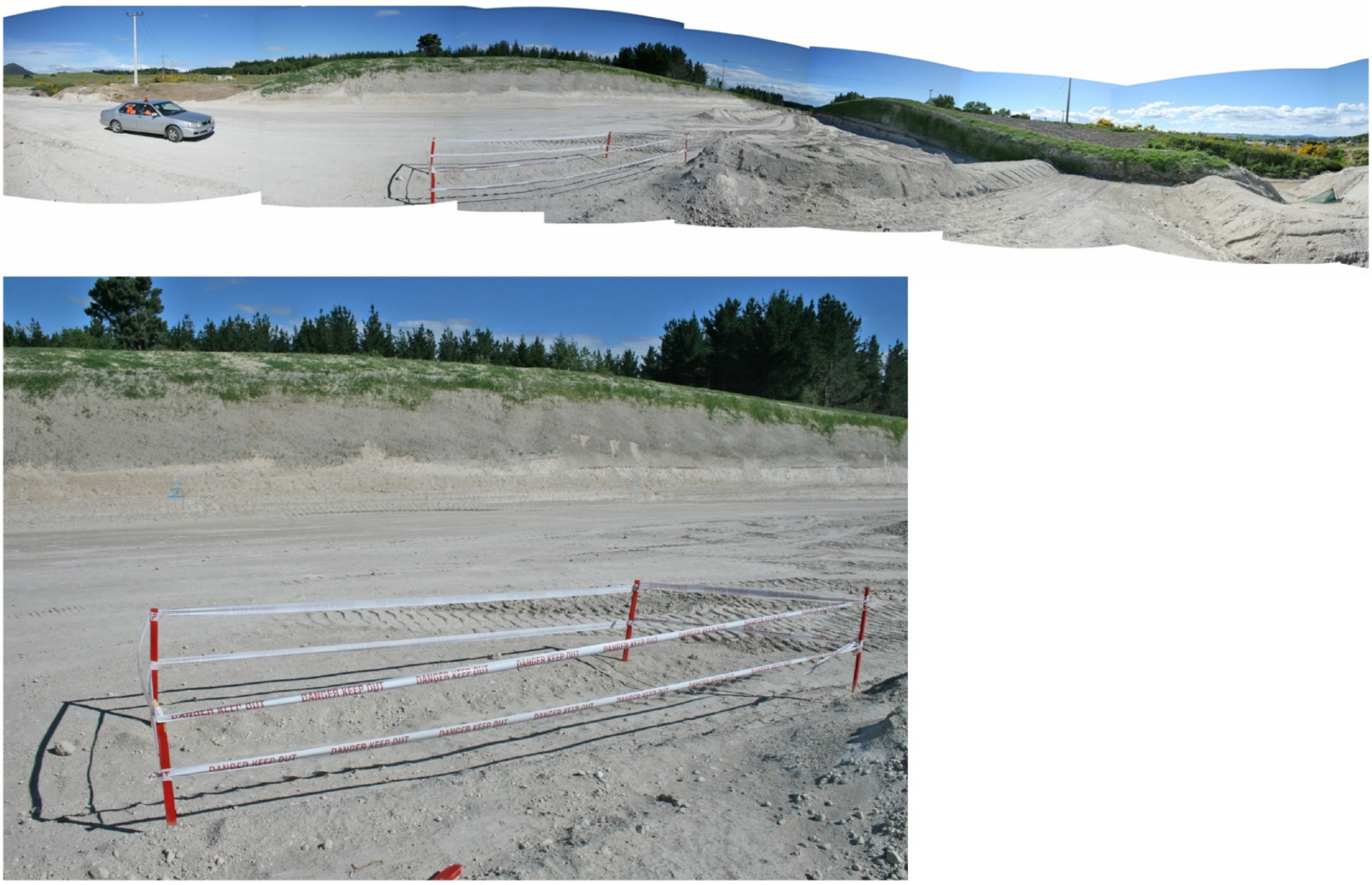

Supplementary Figure 3 Panoramic (above) and detail view looking south (below) of site of Taupo Bypass moa deposit (38° 43' 20.111" S, 176° 06' 03.817" E; c. 440 m a.s.l.), 12 Novermber 2009. The birds had been trapped in a cavity (outlined by the tapes) within the Taupo Ignimbrite in which the new road bed is being developed. Minimum depth of ignimbrite shown in section at rear. The deposit was discovered on or before 9 November 2009, during excavations by heavy machinery.

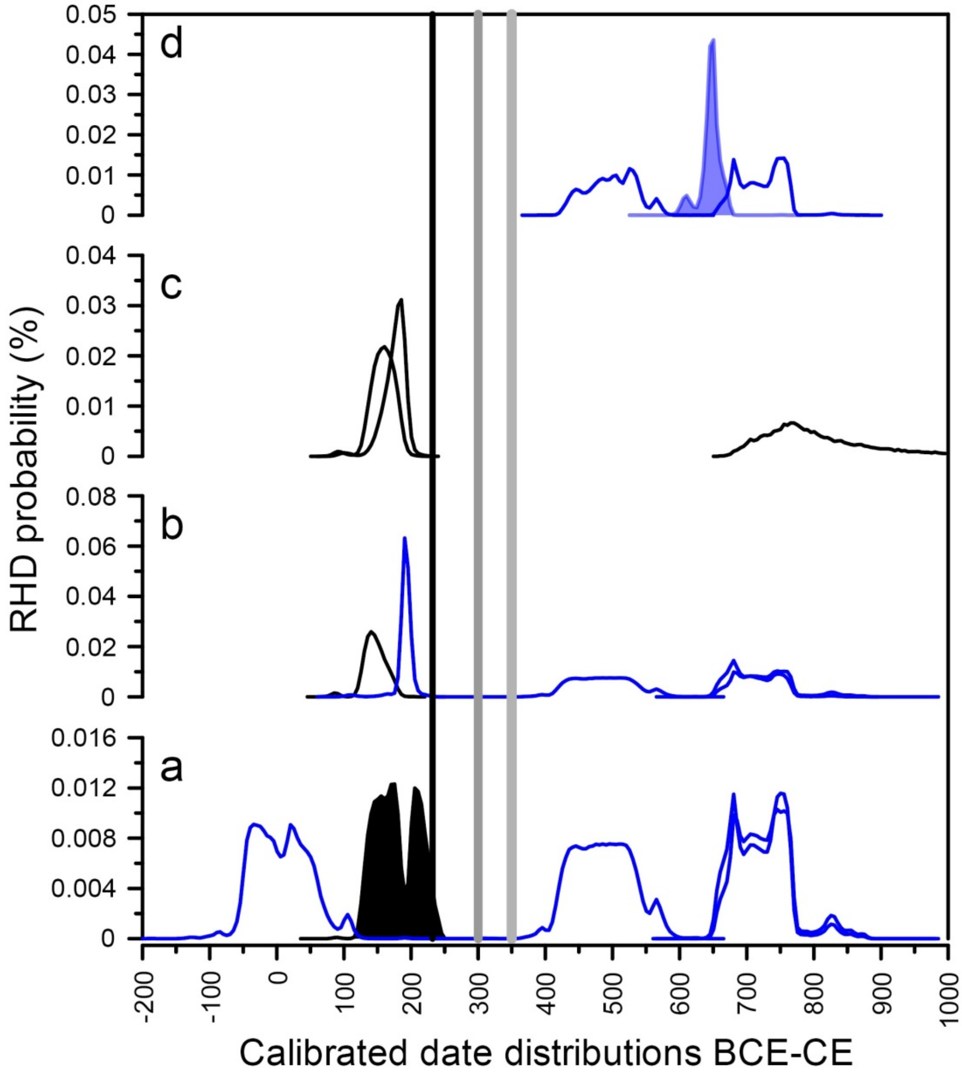

Supplementary Figure 4 Calibrated age distributions for radiocarbon ages of four female *Euryapteryx curtus* recovered from a cavity in the Taupo eruption ignimbrite, and therefore post-dating the eruption, in comparison with the age measured on the outer rings of the wiggle match tree (WK23140). Black vertical line: wiggle match age, 232 CE; central line, minimum date of vegetation post-wiggle match date for eruption; right hand line, probable date for vegetation recovered sufficiently for support moa. **a**, WK23140, black, filled distribution; blue lines, unmodelled calibrated date distributions for moa (left to right, NZA34021, 34019, 34022, 34020). **b**, modelled distributions from Phase analysis: Phase 1, WK23140, pre-dating eruption; Phase 2, all moa, moa post-dating Taupo ignimbrite. Note that moa date NZA34021 has been forced to post-dating eruption date distribution; analysis generated error warnings for poor agreement with the swapping of ages WK23140 and NZA34021 and between NZA34021 and NZA34019; distributions for NZA34019, 34022, 34020 were not displaced. **c**, distributions of Phase boundaries: left to right, end of Phase 1, start of Phase 2, end of Phase 2. Note that the boundary between Phases 1 and 2 (date of the eruption) is c. 50 years older than the wiggle match date. **d**, probability distributions for combined ages for moa: left to right, all four; youngest three (filled); youngest two.

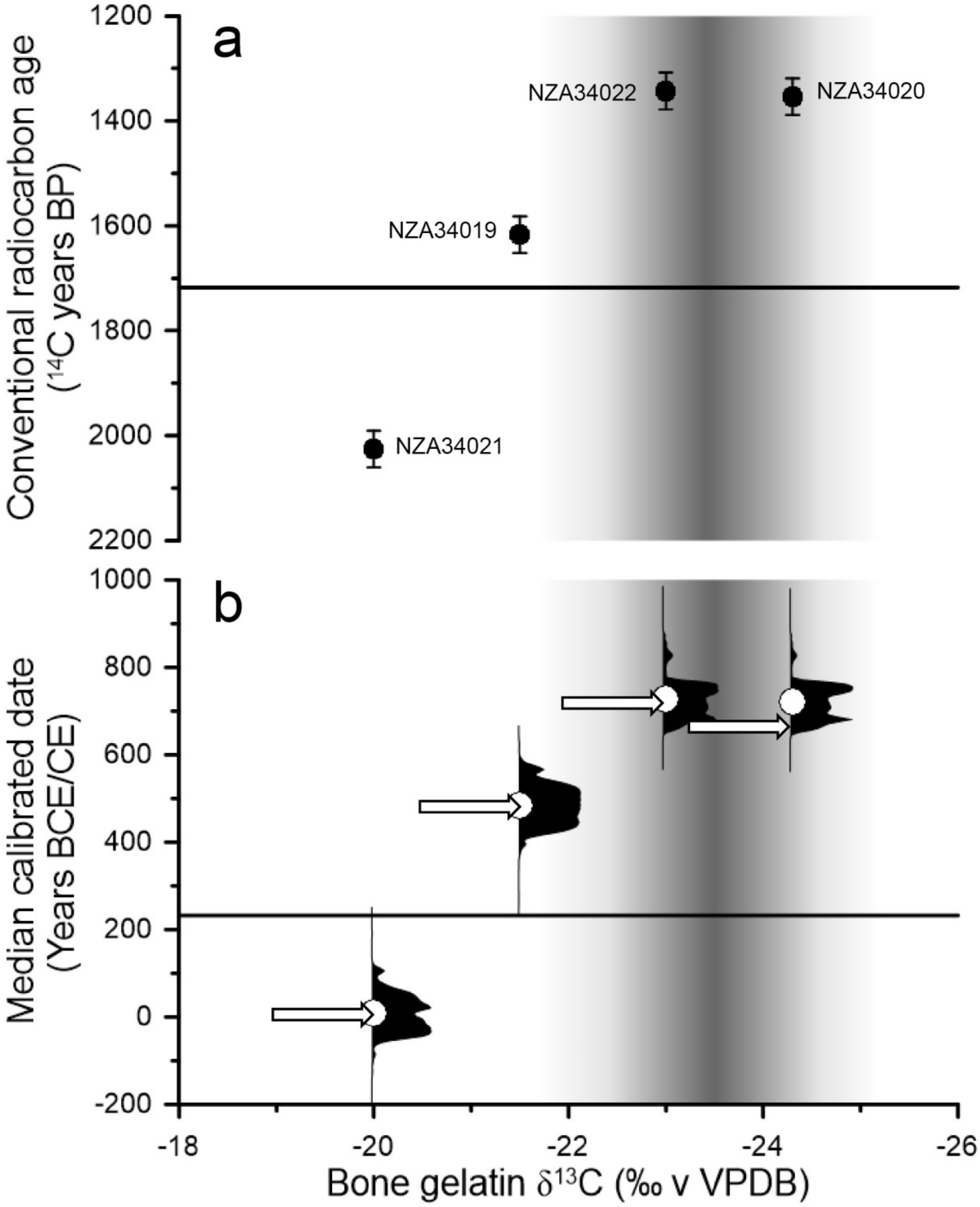

Supplementary Figure 5 Conventional radiocarbon ages (**a**), with 1σ measurement error bars, and their median calibrated dates and probability distributions (**b**) on four female *Euryapteryx curtus* in relation to their bone gelatin $\delta^{13}C$ values. All individuals post-date the ignimbrite eruption. Horizontal lines, wiggle match date for eruption, 232 CE (1718 BP). Shaded area indicates mean ± SD values for 51 $\delta^{13}C$ measurements on *Eu curtus* bone gelatin of Pleistocene and Holocene age, for all New Zealand. Arrows indicate usual ~1‰ offset between vegetation and bone gelatin $\delta^{13}C$ values. The small sample size limits statistical analysis but a Reduced Major Axis regression of conventional radiocarbon ages on $\delta^{13}C$ had a uncorrected *P* value of 0.0644, bordering on significant, with a permutation *P* of 0.0865, for an R^2 value of 0.875; a Generalized Linear Model gave a *P* (slope = 0) of 0.000179.

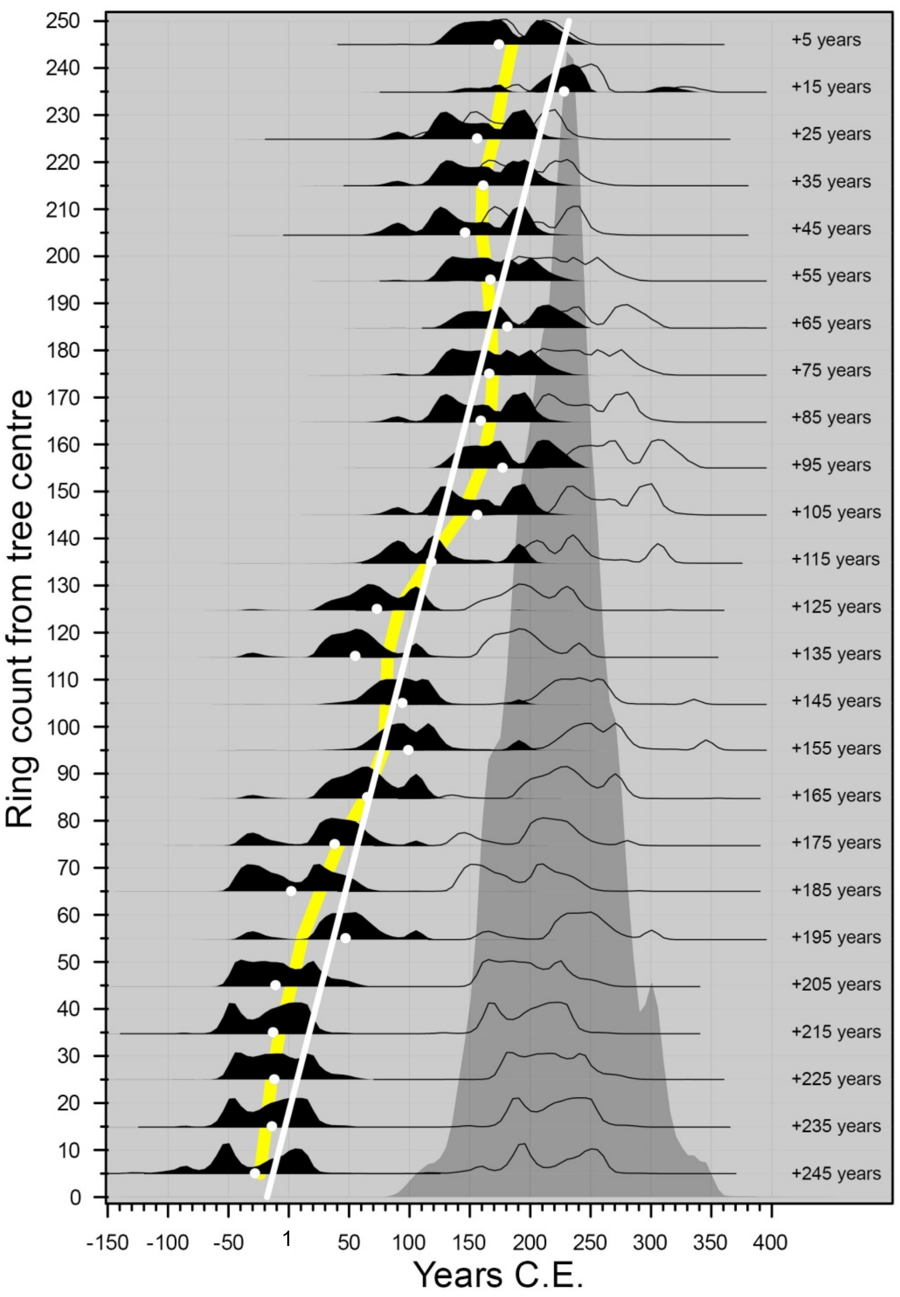

Supplementary Figure 6 Illustration of method of combining calibrated radiocarbon age probability distributions for tree ring sets aligned by actual (from ring counts) differences between dates of laying down of carbon whose ^{14}C content is measured. White line indicates linear change in age of semi-decadal samples. Note deviations (both older and younger) of calibrated probability distributions relative to linear growth sequence.

Supplementary Table 1 Location of radiocarbon-dated samples relative to the Taupo eruption vent, with dated material and source references.

Colour ramps highlight the ubiquitous distance-age relationship, especially for wood and charcoal. Red = old and close, blue = young and far.

Location relative to vent					Median age (SHCal13)			List		
Bearing (degrees)	Distance (km)	Lab no.	CRA	Error	BP	CE	Material	No	Ref. nos.[20]	Rating[20]
130.7	67.4	NZA6636[1]	1775	93	1650	300	Bone gelatin	17		
130.7	67.4	NZA7183[2]	1570	68	1422	528	Bone gelatin	19		
130.7	67.4	NZA8226[2]	1715	69	1763	187	Carbonised leaf	16		
130.7	67.4	NZA7532[2]	1871	67	1580	370	Carbonised leaf	18		
22.03	28.6	NZ82[3,4]	2040	50	1914	36	Charred wood	1	14,8	2
44.6	21.6	NZ176[3,4]	1960	70	1795	155	Charcoal	3	14,8	1
237.4	33.5	NZ3[3,4]	1970	150	1883	67	Charcoal	4	14,8	1
32.5	33.5	NZ175[3,4]	1850	100	1737	213	Charcoal	2	14,8	1
132.32	34	NZ1[3,4]	1820	150	1706	244	Charcoal	5	14,8	1
204.3	44.3	NZ4[3]	1920	150	1823	127	Charcoal	8	14	2
14	44.4	NZ163[3,4]	1840	50	1726	224	Charcoal	9	14,8	1
353.4	45.2	NZ162[3,4]	1830	70	1712	238	Charcoal	10	14,8	1
353.4	45.2	NZ161	1780	80	1655	295	Charcoal	11	14,8	1
7.3	51	NZ174[3,4]	1800	100	1677	273	Charcoal	12	14,8	1
7.3	51	NZ173[3,4]	1750	50	1624	326	Charcoal	13	14,8	1
231.9	59.5	NZ170[3,4]	1800	50	1666	284	Charcoal	15	14,8	1
16.2	68.5	Wk928[5]	1870	60	1764	186	Charcoal	36	f	1
42.5	78.7	NZ1548[6,7,8]	1840	50	1726	224	Charcoal	20	56,7,21	1
37.5	79.9	NZ172[3,4]	1800	100	1667	283	Charcoal	21	14,8	1
111.3	81.1	NZ183[3,4]	1840	70	1673	277	Charcoal	22	14,8	1
141.5	115.8	NZ158[3,4]	1760	80	1634	316	Charcoal	26	14,8	1
335.1	127.1	NZ38[3,4]	1800	70	1673	277	Charcoal	30	14,8	1
332.1	148.9	NZ37[3,4]	1780	60	1652	298	Charcoal	32	14,8	1
339.8	369.6	NZ1764[9]	1805	65	1677	273	Charred wood	35		
332.4	120	Wk215[10-13]	1730	60	1600	350	Gyttja	28	16,25,28,12	2
131.2	104.6	NZ5610A[14]	1790	65	1661	289	Peat/Wood	24	20	2
42.5	122	NZ1059[15-17]	1870	60	1764	186	Peat	29	37,39,9	1
80.9	170.3	NZ502[16,17]	1770	70	1645	305	Peat	33	9,39	1
347.2	179.2	NZ3121[9]	1680	70	1536	414	Peat	34	55	3*
38.7	38.5	NZ168[3,4,18]	1980	40	1794	156	Wood	6	14,8,43	1
12.3	43.9	NZ164[3,4]	1890	70	1784	166	Wood	7	14,8	1
321	51.8	Wk23140[19]	1874	18	1776	174	Wood cellulose	14		
342.9	91.5	NZ165[3,4]	1900	70	1795	155	Wood	23	14,8	1
131.2	104.6	NZ5611A[14]	1735	65	1606	344	Wood	25	20	2
141	115.8	NZ159[3,4]	1750	80	1623	327	Wood	27	14,8	1
80.9	135	NZ524[16,17]	1775	75	1649	301	Wood	31	9,39	1

Notes. *given a "3" rating as a minimum age noted as "?too young" [20]. Dates not included in plot: Wk1015, 1690±80 (rating 1, marked as a "minimum"[20]); Wk1016 1790±80 rating 1, marked as a "maximum"[20]); Wk1094 1800±50 (rating 1, in situ tree trunk in Taupo Pumice alluvium[20]).

References

1 Holdaway, R. N. A spatio-temporal model for the invasion of the New Zealand archipelago by the Pacific rat *Rattus exulans*. *J. R. Soc. N. Z.* **29**, 91-105 (1999).

2 Holdaway, R. N. & Beavan, N. Reliable ^{14}C AMS dates on bird and Pacific rat *Rattus exulans* bone gelatin, from a $CaCO_3$-rich deposit. *J. R. Soc. N. Z.* **29**, 185-211 (1999).

3 Healy, J. Dating of the younger volcanic eruptions of the Taupo region. Part 1. *N. Z. Geol. Surv. Bull.* **73**, 7-39 (1964).

4 Grant-Taylor, T. L. & Rafter, T. A. New Zealand natural radiocarbon measurements I-V. *Radiocarbon* **5**, 118-162 (1963).

5 Unpublished dates: Lowe, D. J. & Hogg, A. G.

6 Pullar, W. A. Tephra marker beds in the soil and their application in related sciences. *Geoderma* **10**, 161-1681973.

7 Goh, K. M.; Pullar, W. A. 1977. Radiocarbon dating techniques for tephras in central North Island, New Zealand. *Geoderma 18*: 265-278.

8 Kennedy, N. M., Pullar, W. A. & Pain, C. F. Late Quaternary land surfaces and geomorphic changes in the Rotorua Basin, North Island, New Zealand. *N. Z. J. Geol. Geophys.* **21**, 249-264 (1978).

9 Pullar, W. A., Kohn, B. P. & Cox, J. E. Airfall Kaharoa Ash and Taupo Pumice, and sea-rafted Loisels Pumice, Taupo Pumice, and Leigh Pumice in northern and eastern parts of the North Island, New Zealand. *N. Z. J. Geol. Geophys.* **20**, 697-717 (1977).

10 Hogg, A. G., Lowe, D. J. & Hendy, C. H. University of Waikato radiocarbon dates 1. *Radiocarbon* **29**, 263-301 (1987).

11 Lowe, D. J. Stratigraphy, age, composition, and correlation of late Quaternary tephras interbedded with organic sediments in Waikato lakes, North Island, New Zealand. *N. Z. J. Geol. Geophys.* **31**, 125-165 (1988).

12 Lowe, D. J., Hogg, A. G., Green, J. D. & Boubée, J. A. T. Stratigraphy and chronology of late Quaternary tephras in Lake Maratoto, Hamilton, New Zealand. *N. Z. J. Geol. Geophys.* **28**, 481-485 (1980).

13 Green, J. D. & Lowe, D. J. Stratigraphy and development of c. 17000 year old Lake Maratoto, North Island, New Zealand, with some inferences about postglacial climatic change. *N. Z. J. Geol. Geophys.* **28**, 675-699 (1985).

14 Hull, A. G. Pre-A.D. 1931 tectonic subsidence of Ahuriri Lagoon, Napier, Hawke's Bay, New Zealand. *N. Z. J. Geol. Geophys.* **29**, 75-82 (1986).

15 Pullar, W.A. Pumice ash beds and peaty deposits of archaeological significance near Lake Poukawa, Hawke's Bay, New Zealand. *N. Z. J.Sci.***13**, 687-705 (1970).

16 Pullar, W. A. & Heine, J. C. in *Ages, inferred from* ^{14}*C ages, of some tephra and other deposits from Rotorua, Taupo, Bay of Plenty, Gisborne, and Hawke's Bay districts*. Radiocarbon Users' Conference, 17-18 August 1971, Lower Hutt, New Zealand (118-138, 1971).

17 Grant-Taylor, T. L. & Rafter, T. A. New Zealand radiocarbon age measurements - 6. *N. Z. J. Geol. Geophys.* **14**, 364-402 (1971).

18 Vucetich, C. G. & Pullar, W. A. 1964. Stratigraphy of Holocene ash in the Rotorua and Gisborne districts. Part 2. *N. Z. Geol. Surv. Bull.* **73**, 43-63.

19 Hogg, A., Lowe, D. J., Palmer, J., Boswijk, G. & Bronk Ramsey, C. Revised calendar date for the Taupo eruption derived by ^{14}C wiggle-matching using a New Zealand kauri ^{14}C calibration data set. *Holocene* **22**, 439-449 (2011).

20 Froggatt, P. C. & Lowe, D. J. A review of late Quaternary silicic and some other tephra formations from New Zealand: Their stratigraphy, nomenclature, distribution, volume, and age. *N. Z. J. Geol. Geophys.* **33**, 89-109 (1990).

Sample	Elemental N (%)	$\delta^{15}N_{AIR}$ (‰)	Elemental C (%)	$\delta^{13}C_{V-PDB}$ (‰)	CN ratio
2	14.48	3.17	41.42	-20.25	3.34
4	13.78	4.40	38.97	-21.74	3.30
Reference Standards					
Standard					
IA-R042 (Bovine liver)		7.63		-21.63	
IA-R042 (Bovine liver)				-21.55	
IA-R042 (Bovine liver)				-21.64	
IA-R042 (Bovine liver)				-21.51	
IA-R042 (Bovine liver)				-21.65	
	Mean	7.59		-21.60	
	SD	0.05		0.06	
	n	5		5	
	Accepted	7.65		-21.60	
Standard					
IA-R045 ($(NH_4)_2SO_4$)		-4.65			
IA-R045 ($(NH_4)_2SO_4$)		-4.77			
	Mean	-4.71			
	SD	0.08			
	n	2			
	Accepted	-4.71			
Standard					
IA-R005 (beet sugar)				-25.90	
IA-R005 (beet sugar)				-26.16	
	Mean			-26.03	
	SD			0.18	
	n			2	
	Accepted			-26.03	
Standard					
IA-R046 ($(NH_4)_2SO_4$)		22.00			
IA-R046 ($(NH_4)_2SO_4$)		22.03			
	Mean	22.02			
	SD	0.02			
	n	2			
	Accepted	22.04			
Standard					
IA-R006 (cane sugar)				-11.76	
IA-R006 (cane sugar)				-11.82	
	Mean			-11.79	
	SD			0.04	
	n			2	
	Accepted			-11.64	

Supplementary Table 2 Carbon and nitrogen stable isotopic ratio measurements on two “oldest” female *Euryapteryx curtus* from the Taupo Bypass site.

**Glenore Matai (*Prumnopitys taxifolia*) Tree 1**			
Sample	$\delta^{13}C_{V\text{-}PDB}$ (‰)	Elemental Carbon (%)	Approx. date CE
GM1_1	-23.5	48.9	1995
GM1_2	-23.6	48.7	1985
GM1_3	-23.5	48.5	1975
GM1_4	-24.0	49.3	1965
GM1_5	-24.1	49.6	1955
GM1_6	-24.0	50.0	1805
GM1_7	-23.6	49.8	1795
GM1_8	-24.0	50.3	1785
GM1_9	-24.0	50.1	1730
GM1A	-25.1	49.1	1936
GM1B	-24.1	49.6	1926
GM1C	-22.6	50.1	1916
GM1D	-22.5	48.7	1906
GM1E	-22.8	49.4	1863
GM1F	-22.9	49.8	1857
GM1G	-23.1	49.9	1844
GM1H	-23.3	49.6	1834
GM1I	-24.2	49.9	1755
Standard	-38.3±0.09 ‰		
**Glenore Matai (*Prumnopitys taxifolia*) Tree 2**			
Sample	$\delta^{13}C_{V\text{-}PDB}$ (‰)	Elemental Carbon (%)	Approx. date CE
GM2_1	-22.7	48.7	1982
GM2_2	-23.2	48.8	1960
GM2_3	-23.7	50.5	1898
GM2_4	-22.8	50.4	1816
GM2_5	-22.8	50.5	1805
GM2_6	-22.5	51.0	1795
GM2_7	-22.7	50.5	1785
GM2_8	-22.8	50.2	1730
GM2_9	-22.8	49.9	1720
GM2_10	-22.9	49.9	1969
GM2_A	-23.8	49.8	1973
GM2_B	-24.9	48.8	1950
GM2_C	-25.1	49.9	1932
GM2_D	-24.1	48.8	1922
GM2_E	-23.8	50.4	1886
GM2_F	-23.7	49.4	1875
GM2_G	-23.5	50.3	1865
GM2_H	-23.4	51.2	1855
GM2_I	-23.2	50.5	1845
GM2ZERO	-24.4	51.1	1745
Standard	-38.3±0.09 ‰		
**Fox River Rimu 1 (*Dacrydium cupressinum*)**			
Sample	$\delta^{13}C_{V\text{-}PDB}$ (‰)	Elemental Carbon (%)	Approx. date CE
FRR1_0	-26.2	47.4	2000
FRR1_50	-25.2	47.0	1950
FRR1_100	-24.5	46.9	1900
FRR1_150	-25.5	49.3	1850
FRR1_200	-24.6	48.3	1800
FRR1_250	-25.5	50.7	1750
FRR1_300	-24.3	49.0	1700
FRR1_350	-25.3	48.5	1650
FRR1_400	-25.5	47.7	1600
FRR1_450	-25.5	47.7	1550
FRR1_500	-27.1	49.2	1500
FRR1_550	-27.1	48.6	1450
FRR1_600	-27.7	48.5	1400
FRR1_636	-28.7	48.9	1360
Standard	-38.3±0.09 ‰		

Supplementary Table 3 Carbon stable isotopic ratio measurements on matai (*Prumnopitys spicatus*) and rimu (*Dacrydium cupressinum*) tree wood.
